# Microsatellite markers for *Anthericum ramosum*: Development, characterization, and cross‐species amplification

**DOI:** 10.1002/aps3.11323

**Published:** 2020-02-14

**Authors:** Petr Vít, Karol Krak, Jan Douda, Bohumil Mandák

**Affiliations:** ^1^ Faculty of Environmental Sciences Czech University of Life Sciences Prague Kamýcká 129 Praha 6–Suchdol CZ‐165 00 Czech Republic; ^2^ The Czech Academy of Sciences Institute of Botany Zámek 1 Průhonice CZ‐252 43 Czech Republic

**Keywords:** Agavaceae, *Anthericum ramosum*, cross‐species amplification, microsatellites

## Abstract

**Premise:**

A set of polymorphic nuclear microsatellite loci was developed and tested for use in population genetic analyses of *Anthericum ramosum* (Agavaceae) and related species.

**Methods and Results:**

Sequences of 110 primers were extracted in silico from Illumina MiSeq genome skimming data. The degree of polymorphism of 19 loci was tested in four *A*. *ramosum* populations collected in Central and Eastern Europe. The average number of alleles per loci ranged from two to 17, and levels of observed and expected heterozygosity ranged from 0.000 to 1.000 and from 0.100 to 0.900, respectively. Primers were successfully amplified in the closely related species *A. liliago* (12 loci) and *Chlorophytum comosum* (six loci), whereas they mostly failed to amplify in the phylogenetically more‐distant species *Muscari comosum* (three loci) and *M*. *tenuiflorum* (no amplification).

**Conclusions:**

This newly developed set of polymorphic nuclear microsatellite markers will be useful for population genetic investigation of *A*. *ramosum* and closely related species.

The genus *Anthericum* L. (Agavaceae) comprises more than 50 perennial herbaceous species, distributed mainly in Africa and Central and South America. Three species, all within section *Anthericum*, are native to Europe, namely *A. baeticum* Boiss., *A. liliago* L., and *A. ramosum* L. (Hegi, 1939). The European species have different ecologies: *A*. *baeticum* is found in wet meadows of the Iberian Peninsula, whereas *A*. *liliago* and *A*. *ramosum* are found in dry grasslands (Hegi, 1939; Rosquist, 2001). All three European *Anthericum* species are morphologically similar, with their main discriminating characters present in their inflorescences (Hegi, 1939).

*Anthericum ramosum* is a perennial diploid (2*n* = 30, 32) species that is self‐compatible and insect‐pollinated with frequent clonal reproduction (Rosquist, 2001). This species has a wide European distribution from Spain to western Russia (Meusel et al., 1965) and is an important component of sub‐Mediterranean (Pontic) dry steppe habitats (Chytrý, 1995). It occurs in open, xeric, and sunny slopes and dry open forests with elevated CaCO_3_ contents, from lowlands to ca. 1800 m in Central Europe (Hegi, 1939). Although the ecology of the species is similar to the tetraploid *A*. *liliago*, which prefers more stony slopes and rocks with predominantly southern exposure, these species co‐occur only rarely (Rosquist, 2001). Mixed populations with the occurrence of hybrid individuals have been documented in Scandinavia (Rosquist, 2001).

The Central European distribution of *A*. *ramosum* is discontinuous, with populations growing among fragmented dry steppe–like habitats. The current distribution pattern is mainly the result of past changes in agricultural practice, which have resulted in significant loss of continuous dry steppe habitats. Knowledge of genetic variation in fragmented populations of this species may serve as a stepping stone to better understand its historical origins and past migration patterns. It may also reveal the population genetic processes taking place in fragmented populations of other important steppe species, and thus offer insights into processes that may endanger these fragmented populations (e.g., loss of genetic diversity caused by genetic drift).

## Methods and Results

### Microsatellite development

DNA of *A. ramosum* was extracted from silica gel–dried leaves taken from a single plant using the DNeasy Plant Mini Kit (QIAGEN, Hilden, Germany), according to the manufacturer's protocol. The DNA library was prepared with a NEBNext Ultra II DNA Library Prep Kit for Illumina (New England Biolabs, Ipswich, Massachusetts, USA), and it was sequenced on the Illumina MiSeq platform using the MiSeq Reagent Kit (2 × 300 bp; Illumina, San Diego, California, USA), together with another nine indexed libraries, in one run. Sequencing resulted in 1,677,883 raw reads (available at the National Center for Biotechnology Information [NCBI] Sequence Read Archive [accession no. SRR10054461]). Chloroplast reads were removed with Bowtie 2 (Langmead and Salzberg, 2012) using the reference chloroplast sequence of *A*. *ramosum* (GenBank accession no. KX790364.1). Sequences containing perfect microsatellite motifs (i.e., di‐, tri‐, and tetranucleotide repeats with minimum lengths of 14, 18, and 20 bp, respectively) were extracted using SSR_pipeline (Miller et al., 2013). Primers were designed for all extracted sequences using Primer3 (Untergasser et al., 2012), as integrated in MSATCOMMANDER version 0.8.2 (Faircloth, 2008). See Appendix 1 for settings of programs and scripts used for manipulation with sequence reads.

### Biological validation

A total of 110 randomly selected candidate primer pairs (defining repeats with 100–400 bp amplicon lengths) were tested for amplification in seven individuals of *A*. *ramosum* from different populations (Appendix 2). Total genomic DNA of *A. ramosum* was extracted from silica gel–dried leaves using the DNeasy 96 Plant Kit (QIAGEN). PCR was carried out in volumes of 10 μL using the QIAGEN Multiplex PCR Kit. The reaction contained 1× concentrated QIAGEN Multiplex PCR Master Mix, 0.05 μM of forward and 0.2 μM of reverse primer, and 10 ng of genomic DNA. The PCR fragments were fluorescently labeled by adding 0.2 μM M13 primer (with either FAM, NED, VIC, or PET; Thermo Fisher Scientific, Waltham, Massachusetts, USA) in the reaction as described in Schuelke (2000). The following PCR profile was used: an initial denaturation step at 95°C for 15 min; followed by 25 cycles of denaturation at 95°C for 30 s, annealing at 55°C for 30 s, and extension at 72°C for 2 min; followed by 10 cycles of denaturation at 95°C for 30 s, annealing at 50°C for 30 s, and extension at 72°C for 2 min; with a final extension at 72°C for 10 min. The success of amplification was verified by electrophoresis in a 2% agarose gel, with only 79 primer pairs showing clear bands for the seven individuals used for the polymorphism tests. One microliter of PCR product was added to a mix of 12.0 μL of Hi‐Di Formamide (Applied Biosystems, Waltham, Massachusetts, USA) and 0.2 μL of GeneTrace LIZ 500 Size Standard (Carolina Biosystems, Prague, Czech Republic) for fragment analysis on the Applied Biosystems 3500 Genetic Analyzer. The electropherograms were analyzed using GeneMarker version 2.7.4 (SoftGenetics, State College, Pennsylvania, USA). Finally, 19 easily scorable, polymorphic markers were selected for genetic analysis of four *A*. *ramosum* populations and for a cross‐species amplification test of *A. liliago*,* Chlorophytum comosum* (Thunb.) Jacques, *Muscari comosum* (L.) Mill., and *M. tenuiflorum* Tausch (Table 1, Appendix 2).

**Table 1:** Characteristics of 19 polymorphic microsatellite loci developed in *Anthericum ramosum*.^a^

Locus	Primer sequences (5′–3′)	Repeat motif	Allele size range (BP)	Fluorescent dye^b^	GenBank accession no.
AR‐di‐05	F: CCGCAAACCTAGCAGTAGAG	(AC)_14_	181–193	6‐FAM^m1^	MN259494
	R: TGATTCTCATTTGACCGGCC				
AR‐di‐12	F: GTTGAGGTTGAGGTGCGATG	(AT)_12_	302–322	6‐FAM^m1^	MN259502
	R: AGGAGAGGAGGTAAAGTCGC				
AR‐di‐27	F: TGATCTTAAACTGTGCGGCC	(AG)_25_	382–420	6‐FAM^m2^	MN259492
	R: GTCACATTGTTCTGGCCACC				
AR‐di‐33	F: GGGACCCTTGTTTGGATGTG	(AG)_15_	204–248	VIC^m2^	MN259497
	R: CAGACAATACGGATGGCGTC				
AR‐di‐34	F: TTCTTTGACGGAACCTCTGC	(AG)_19_	152–186	PET^m2^	MN259498
	R: GAAACAGAGCGCAGGGAAG				
AR‐di‐50	F: ACCAACATTTCACAAGGCAAC	(AC)_20_	231–263	PET^m1^	MN259503
	R: ACACCTCCTACTCAGAACCG				
AR‐di‐96	F: CCCTCACATTTCTTCGTAACAC	(AG)_8_	250–258	6‐FAM^m1^	MN259493
	R: AAGTGCAGCAGGTTGAAACG				
AR‐di‐98	F: CACGCCGTATCATTCCCTTC	(AG)_20_	213–257	6‐FAM^m2^	MN259495
	R: GAACTTGTTTCCGGCCGATC				
AR‐di‐101	F: CACTCGCACTCTTGTAGCAG	(AG)_21_	187–251	VIC^m1^	MN259496
	R: GCTGTCACTACACTAGACTTGG				
AR‐di‐106	F: CTCCCATCTTCTCACTACGC	(AG)_9_	147–175	PET^m1^	MN259499
	R: TCATCCATTAGCCCTCCTCC				
AR‐di‐107	F: CATCAGAACCCGAGCAATCC	(AC)_7_	202–238	NED^m2^	MN259500
	R: TGGGAGAGCAACATTTCCATC				
AR‐di‐108	F: TTGTATTTGTCGTGCCGTCG	(AG)_7_	134–146	VIC^m1^	MN259501
	R: GGAGAACGCTGGCAAGATTC				
AR‐tet‐65	F: TACACACGTACATGCTCGGC	(ACGC)_6_	110–128	VIC^m2^	MN259505
	R: GGTCTCTGCCAAATGTGTGC				
AR‐tet‐91	F: CGAGTCAGTATCTTCCGTCAC	(AAAT)_5_	247–267	NED^m1^	MN259504
	R: ACGAGGTTCCCGAAGATGTC				
AR‐tri‐16	F: CACCCTATCACTCCTCAAACAC	(AAG)_11_	232–338	PET^m2^	MN259506
	R: GTGCTGGGTTGATTGCTCTC				
AR‐tri‐59	F: AAACTCCTCCATCATCCCATTC	(AAG)_10_	258–285	PET^m2^	MN259509
	R: TTATCCCACATGTCAGCGGG				
AR‐tri‐60	F: TCTCTGCTACATTCAAGTTGGC	(AAT)_11_	406–430	VIC^m1^	MN259510
	R: GGTCACCCGGGTAACTCAC				
AR‐tri‐86	F: GCTTGAGTGATTTGTTGCGC	(AAT)_10_	329–386	NED^m2^	MN259507
	R: GGCAATGCATAAAGGTTCAGC				
AR‐tri‐90	F: ATGGACTACTCTCAGCAGGC	(ATC)_8_	134–146	6‐FAM^m1^	MN259508
	R: GCATGCGTAGGGAACATAGC				

a An M13 tag (GGAAACAGCTATGACCAT; Schuelke, 2000) was added to the 5′ end of the forward primer. Optimal annealing temperature was 55°C.

b The multiplex number is indicated as m1 or m2.

### Microsatellite data analysis and results

For each polymorphic microsatellite locus, the following basic genetic diversity parameters were calculated by using the package diveRsity in R (Keenan et al., 2013): the number of alleles (*A*), levels of observed (*H*_o_) and expected (*H*_e_) heterozygosities, and the inbreeding index *f* (*F*_IS_) (Weir and Cockerham, 1984) as a measure of departure from within‐population random mating. The presence of null alleles was evaluated in MICRO‐CHECKER 2.2.3 (van Oosterhout et al., 2004). Deviation from Hardy–Weinberg equilibrium was calculated using Fisher's exact test for each locus, and *P* values were adjusted using Bonferroni correction (Wright, 1992).

The basic genetic parameters of four populations are presented in Table 2. A total of 195 alleles were identified in the 19 polymorphic loci analyzed. Among the four populations, the number of alleles per locus ranged from two to 17. Levels of heterozygosities (*H*_o_ and *H*_e_) ranged from 0.000 to 1.000 and from 0.100 to 0.900, respectively. The level of inbreeding (inbreeding coefficient *F*_IS_) ranged from –0.504 to 1.000. Two pairs of loci (AR‐di‐101 × AR‐di‐107, AR‐di‐108 × AR‐tri‐90) were significantly linked, probably because of the limited number of individuals and populations. Significant deviation from Hardy–Weinberg equilibrium was detected in 11 out of 19 loci, caused by heterozygosity deficiency in particular populations (see Table 2 for details). Four loci (AR‐tri‐16, AR‐tri‐59, AR‐tri‐60, and AR‐tri‐96) showed significant presence of null alleles (see Table 2). These loci were not further included in the cross‐species amplification tests.

**Table 2:** Basic genetic parameters of the 19 newly developed polymorphic microsatellites of *Anthericum ramosum* tested in four populations.^a^

Locus	AR1 (*N* = 20)					AR3 (*N* = 20)					AR11 (*N* = 20)					AR61 (*N* = 16)					Overall				
	*A*	*H*_o_	*H*_e_	*F*_IS_	HWE^b^	*A*	*H*_o_	*H*_e_	*F*_IS_	HWE^b^	*A*	*H*_o_	*H*_e_	*F*_IS_	HWE^b^	*A*	*H*_o_	*H*_e_	*F*_IS_	HWE^b^	*A*	*H*_o_	*H*_e_	*F*_IS_	HWE^b^
AR‐di‐5	6	0.740	0.740	0.006	ns	4	0.800	0.610	–0.309	ns	6	0.800	0.750	–0.061	ns	7	0.640	0.750	0.146	ns	7	0.750	0.760	0.003	ns
AR‐di‐12	7	0.400	0.770	0.483	*	7	0.290	0.600	0.510	*	6	0.420	0.710	0.410	ns	6	0.470	0.740	0.373	ns	13	0.390	0.780	0.493	*
AR‐di‐27	3	0.350	0.410	0.149	ns	3	0.070	0.570	0.883	*	7	0.370	0.540	0.320	ns	4	0.330	0.300	–0.128	ns	8	0.290	0.490	0.411	*
AR‐di‐33	17	0.840	0.900	0.060	ns	11	0.800	0.810	0.008	ns	14	0.900	0.870	–0.034	ns	11	0.920	0.890	–0.040	ns	21	0.860	0.910	0.057	ns
AR‐di‐34	12	0.700	0.870	0.194	*	11	0.600	0.880	0.320	*	11	0.850	0.850	0.004	ns	13	0.810	0.870	0.067	ns	17	0.740	0.910	0.186	ns
AR‐di‐50	12	0.800	0.870	0.083	ns	9	0.530	0.790	0.332	*	11	0.600	0.830	0.274	ns	9	0.810	0.820	0.014	ns	15	0.680	0.900	0.245	*
AR‐di‐96	3^c^	0.220	0.520	0.570	ns	2^c^	0.070	0.190	0.627	ns	2^c^	0.080	0.470	0.822	ns	2^c^	0.000	0.460	1.000	NA	3	0.100	0.450	0.768	*
AR‐di‐98	11	0.680	0.810	0.157	*	9	0.850	0.870	0.027	ns	12	0.700	0.790	0.118	ns	8	0.880	0.790	–0.103	ns	17	0.770	0.860	0.102	ns
AR‐di‐101	10	0.580	0.830	0.299	ns	8	0.800	0.800	0.005	ns	13	0.680	0.890	0.235	ns	9	0.470	0.760	0.388	ns	14	0.640	0.880	0.271	ns
AR‐di‐106	5	0.650	0.680	0.042	ns	4	0.400	0.560	0.287	ns	5	0.500	0.730	0.315	*	5	0.620	0.690	0.096	ns	5	0.540	0.740	0.272	*
AR‐di‐107	10	0.560	0.820	0.326	ns	8	0.800	0.800	0.005	ns	11	0.600	0.890	0.327	ns	9	0.550	0.750	0.271	*	14	0.640	0.870	0.275	ns
AR‐di‐108	3	0.100	0.100	–0.039	ns	2	0.160	0.150	–0.086	ns	3	0.400	0.430	0.078	ns	2	0.140	0.130	–0.077	ns	4	0.210	0.230	0.116	ns
AR‐tet‐65	6	1.000	0.820	–0.226	ns	5	0.600	0.630	0.048	ns	5	0.600	0.730	0.174	ns	3	0.470	0.500	0.075	ns	6	0.680	0.740	0.083	ns
AR‐tet‐91	3	0.450	0.500	0.102	ns	2	0.750	0.500	–0.504	ns	3	0.600	0.540	–0.108	ns	2	0.190	0.260	0.289	ns	4	0.510	0.500	−0.031	ns
AR‐tri‐16	3^c^	0.270	0.520	0.485	ns	4^c^	0.210	0.680	0.689	*	4^c^	0.120	0.660	0.822	*	4	0.360	0.500	0.289	ns	5	0.230	0.650	0.645	*
AR‐tri‐59	5^c^	0.470	0.690	0.315	ns	3^c^	0.050	0.580	0.914	*	5^c^	0.280	0.730	0.620	*	5^c^	0.200	0.520	0.614	ns	8	0.240	0.690	0.647	*
AR‐tri‐60	7^c^	0.360	0.810	0.560	*	6^c^	0.210	0.690	0.694	*	5^c^	0.380	0.740	0.495	ns	4^c^	0.170	0.670	0.751	*	9	0.280	0.800	0.651	*
AR‐tri‐86	13	0.850	0.900	0.054	ns	10	0.630	0.800	0.206	ns	13	0.900	0.870	–0.032	ns	11	0.640	0.850	0.246	*	21	0.770	0.900	0.149	ns
AR‐tri‐90	3	0.100	0.100	–0.039	ns	2	0.150	0.140	–0.081	ns	3	0.400	0.430	0.078	ns	2	0.120	0.120	–0.067	ns	4	0.200	0.220	0.120	ns

Note

*A* = number of alleles; *F*_IS_ = inbreeding index; *H*_e_ = expected heterozygosity; *H*_o_ = observed heterozygosity; HWE = deviation from Hardy–Weinberg equilibrium after Bonferroni correction; *N* = number of individuals sampled.

a Locality and voucher information are provided in Appendix 2.

b Significant deviation from HWE after Bonferroni correction (*P < 0.0026), ns = not significant.

c Significant presence of null alleles.

Cross‐species amplification was tested in four related species: *A. liliago*,* C. comosum*,* M. comosum*, and *M*. *tenuiflorum*. Amplification of developed markers was partly successful in closely related species (i.e., *A*. *liliago* and *C*. *comosum*) and mostly unsuccessful in phylogenetically more distant species (i.e., *M*. *comosum* and *M*. *tenuiflorum*) (Table 3). Ten markers showed significant variation within *A*. *liliago* individuals; these markers may be suitable for study of populations in which both species occur, as well as for study of gene flow between both species.

**Table 3:** Results of cross‐species amplification of 19 microsatellite markers developed for *Anthericum ramosum* and tested in four related species.^a^

Locus	*Anthericum liliago* (*N* = 5)	*Chlorophytum comosum* (*N* = 1)	*Muscari comosum* (*N* = 5)	*Muscari tenuiflorum* (*N* = 5)
AR‐di‐05	183	—	—	—
AR‐di‐12	—	—	—	—
AR‐di‐27	386–412	276–296	—	—
AR‐di‐33	204–246	—	—	—
AR‐di‐34	—	166	160–226	—
AR‐di‐50	251–253	—	—	—
AR‐di‐98	219–233	221	—	—
AR‐di‐101	—	—	—	—
AR‐di‐106	—	—	—	—
AR‐di‐107	170–242	—	210	—
AR‐di‐108	—	118	—	—
AR‐tet‐65	124–152	—	—	—
AR‐tet‐91	251–259	268	—	—
AR‐tri‐16	311–314	—	—	—
AR‐tri‐59	270	—	—	—
AR‐tri‐60	—	—	—	—
AR‐tri‐86	329–338	—	329	—
AR‐tri‐90	134–146	188–197	—	—
AR‐tri‐96	—	—	—	—

Note

— indicates no amplification; *N* = number of individuals sampled.

a Localities for related species are given in Appendix 2. Allele size range is given if the marker was polymorphic.

## Conclusions

Nineteen novel nuclear microsatellite markers were developed for *A. ramosum*. It is the first set of microsatellites developed for this genus. These markers will be helpful for population genetic studies to reveal phylogeographic patterns of important steppe species. Several markers were successfully amplified within related species, and after optimization, they may be useful in these species for studies of phylogeography, hybridization, or gene flow.

## Author Contributions

P.V., K.K., and B.M. conceived and designed the study. P.V., K.K., J.D., and B.M. collected the plant material. P.V. designed and supervised the laboratory work. P.V. and B.M. analyzed the data and drafted the manuscript.

## Data Availability

The raw sequence reads are deposited in the National Center for Biotechnology Information (NCBI; GenBank Sequence Read Archive accession no. SRR10054461). Information about plant material is stored as BioSample no. SAMN12655405 under BioProject no. PRJNA562837. Sequences of reads used for primer design were uploaded to NCBI, see Table 1 for accession numbers.

## Appendix 1 The in silico SSR development workflow used in this study.

1. Initial sequence trimming using Trimmomatic 0.36 (Bolger et al., 2014). Paired‐end reads were first trimmed with the following settings:

ILLUMINACLIP:adapters_used.fa:2:30:10

LEADING:20

TRAILING:20

SLIDINGWINDOW:4:20

MINLEN:48

2. Removal of chloroplast reads using Bowtie 2 (Langmead and Salzberg, 2012).

A Bowtie index was built from a set of reference DNA sequence (*Anthericum ramosum*, GenBank accession no. KX790364.1) using a bowtie2‐build indexer. Reads of *A. ramosum* were mapped on the reference index using the Bowtie2 aligner with default settings.

3. Extraction of reads containing microsatellites using SSR_pipeline (Miller et al., 2013).

Reads containing microsatellite motifs were extracted using the built‐in commands quality_sort, joinseqs, and SSR_search using default settings. Only the following changes in search parameters were made to extract perfect di‐, tri‐, and tetranucleotide repeats:

dinucleotide repeats: minimum length 14 bp

trinucleotide repeats: minimum length 18 bp

tetranucleotide repeats: minimum length 20 bp

4. Design of primers for all sequences containing the above‐mentioned microsatellite motifs using Primer3 (Untergasser et al., 2012), as integrated in MSATCOMMANDER version 0.8.2 (Faircloth, 2008) with default settings.

## Appendix 2 Locality and voucher information for populations used in this study.^a^

**Table:**

Species	Population code	Voucher no.	*N*	Collection locality	Geographic coordinates	Elevation (m)
*Anthericum ramosum* L.	AR1^b^	BRNU665930	20	Czech Republic, Louny	50.410224°N, 13.807042°E	475
*Anthericum ramosum*	AR3^b^	BRNU667304	20	Poland, Raclawice	50.338267°N, 20.233317°E	300
*Anthericum ramosum*	AR9^b^	BRNU667337	1	Hungary, Eger	48.012401°N, 20.578951°E	590
*Anthericum ramosum*	AR11^b^	BRNU667235	20	Romania, Suceava	47.574910°N, 26.255420°E	325
*Anthericum ramosum*	AR61^b^	BRNU667211	16	Ukraine, Bovshiv	49.223650°N, 24.698897°E	260
*Anthericum liliago* L.	AL28	BRNU667317	5	Germany, Könnern	51.654783°N, 11.756253°E	100
*Chlorophytum comosum* (Thunb.) Jacques “Atlantic”	CC1		1	Commercial cultivar	—	
*Muscari comosum* (L.) Mill.	MC205	BRNU667297	5	Czech Republic, Lhánice	49.108932°N, 16.240021°E	375
*Muscari tenuiflorum* Tausch	MT2	BRNU667112	5	Bulgaria, Pazardzhik	42.125886°N, 24.387949°E	400

Note

*N* = number of individuals sampled.

a Herbarium vouchers are deposited at the herbarium of the Department of Botany and Zoology, Masaryk University, Brno (BRNU), Czech Republic.

b Populations used for the initial PCR amplification test.
